# Supplementary material for: Splice-Junction-Based Mapping of Alternative Isoforms in the Human Proteome
Source: Cell Rep. Author manuscript; Available in PMC 2020 Jan 15. (PMC6961840; doi:10.1016/j.celrep.2019.11.026)
Supplement: 3 [file NIHMS1546469-supplement-3.zip › DF2/PXD006675/LeftAtrium-69-Q0ZGT2-AEQEGDDSLLITVVPVK.pdf]

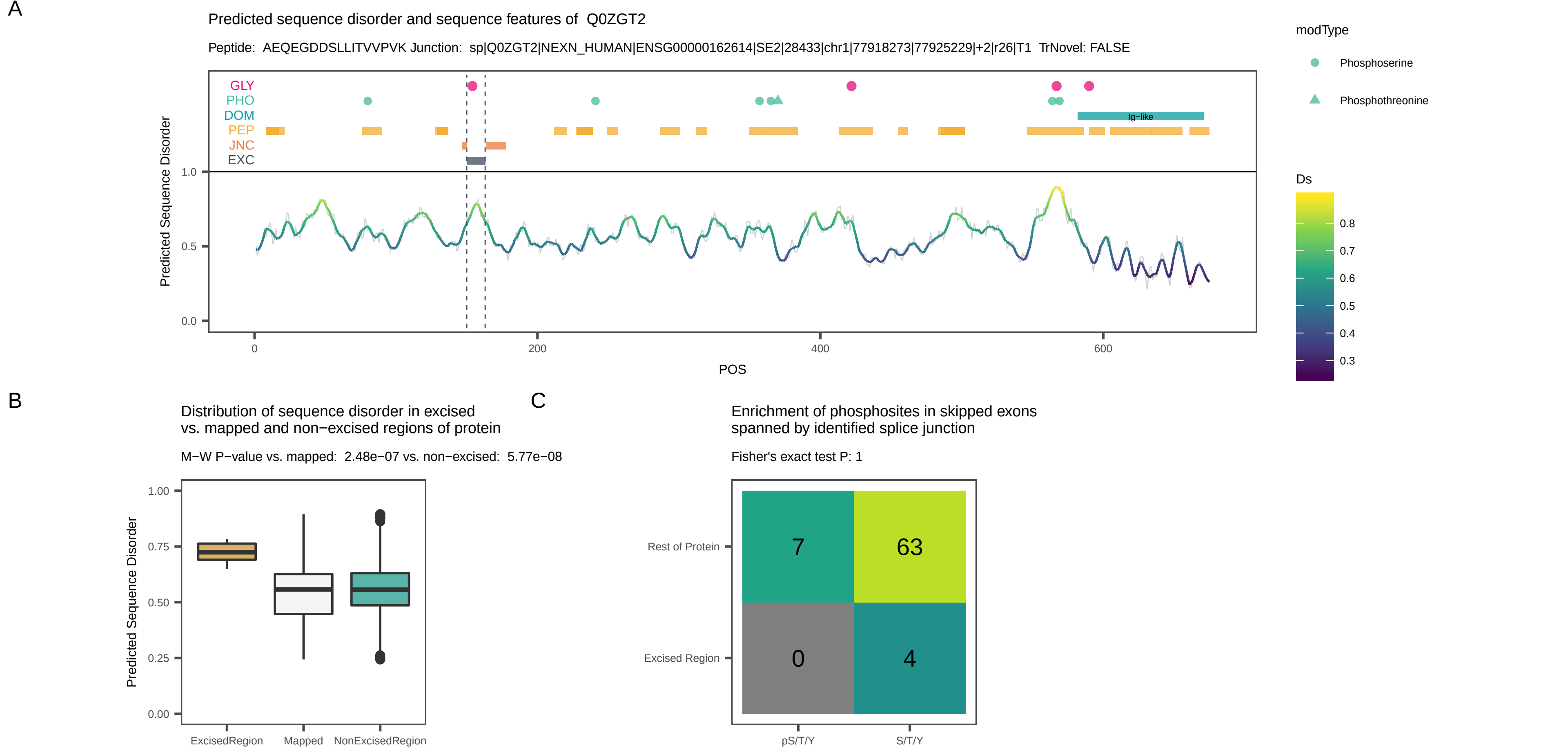
A
Predicted sequence disorder and sequence features of Q0ZGT2
Peptide: AEQEGDDSLLITVVPVK Junction: sp|Q0ZGT2|NEXN_HUMAN|ENSG00000162614|SE2|28433|chr1|77918273|77925229|+2|r26|T1 TrNovel: FALSE
GLY
PHO
DOM
PEP
JNC
EXC
Ig−like
Predicted Sequence Disorder
1.0
0.5
0.0
0
200
400
600
POS
modType
Phosphoserine
Phosphothreonine
Ds
0.8
0.7
0.6
0.5
0.4
0.3
B
Distribution of sequence disorder in excised
vs. mapped and non−excised regions of protein
M−W P−value vs. mapped: 2.48e−07 vs. non−excised: 5.77e−08
Predicted Sequence Disorder
1.00
0.75
0.50
0.25
0.00
ExcisedRegion
Mapped
NonExcisedRegion
C
Enrichment of phosphosites in skipped exons
spanned by identified splice junction
Fisher's exact test P: 1
Rest of Protein
Excised Region
7
63
0
4
pS/T/Y
S/T/Y
